# Fujian Province β-Thalassemia: A Molecular and Hematological Study in Southeastern China

**DOI:** 10.1155/genr/8862095

**Published:** 2025-06-08

**Authors:** Junhao Zheng, Meihuan Chen, Siwen Zhang, Aixiang Lv, Min Zhang, Lingji Chen, Na Lin, Liangpu Xu, Hailong Huang

**Affiliations:** ^1^The School of Medical Technology and Engineering, Fujian Medical University, Fuzhou, China; ^2^Key Laboratory of Clinical Laboratory Technology for Precision Medicine (Fujian Medical University), Fujian Province University, Fuzhou, China; ^3^Medical Genetic Diagnosis and Therapy Center of Fujian Maternity and Child Health Hospital College of Clinical Medicine for Obstetrics and Gynecology and Pediatrics, Fujian Provincial Key Laboratory of Prenatal Diagnosis and Birth Defect, Fujian Medical University, Fuzhou, China

**Keywords:** β-thalassemia, gene frequency, gene mutation, hematological parameters

## Abstract

**Background:** This study aims to investigate the mutation spectrum of β-thalassemia in Fujian Province, China, and to comprehensively analyze the correlation between age, gender, genotype, and hematological parameters in carriers of β-thalassemia.

**Methods:** Genotypes of 10,350 subjects suspected of having thalassemia were analyzed using reverse dot blotting (RDB) or β-globin gene sequencing. Their hematological indices were analyzed by genotype, gender, and age.

**Results:** Among the subjects, 1214 (11.73%) were identified as β-thalassemia carriers. The prevalent genotypes included IVS-II-654 (C > T)/N (37.56%), CD 41-42 (-TTCT)/N (30.72%), CD 17 (A > T)/N (9.64%), −28 (A > G)/N (7.00%), CD 27-28 (+C)/N (3.21%), and CD 26 (GAG > AAG)/N (3.05%). Two rare mutations, Cap+22 (G > A) and IVS-II-806 (G > C), were detected, with the latter being part of a double heterozygous condition with hemoglobin (Hb) New York, compound −α4.2/αα, and Hb Q Thailand, marking the first report in Chinese individuals. Hematological analysis revealed that the CD 26 group exhibited higher levels of Hb, mean corpuscular volume (MCV), and mean corpuscular hemoglobin (MCH) compared to the β^0^ and β^+^ groups (*p* < 0.05). Within the β^+^ group, individuals with −28 (A > G)/N showed significantly higher Hb, MCV, and MCH levels compared to those with IVS-II-654 (C > T)/N. Adult males had higher Hb levels than adult females, and adult patients generally had higher MCV and MCH levels than minors (*p* < 0.05).

**Conclusion:** This study represents the first comprehensive molecular epidemiological investigation and hematological analysis of β-thalassemia in Fujian Province, providing support for the optimization of prevention and control strategies for thalassemia.

## 1. Introduction

Thalassemia is a group of hereditary blood disorders that can seriously threaten human health and result in mortality and disability, which is divided into α-thalassemia (α-thal) and β-thalassemia (β-thal) depending on the globin affected [1]. β-thal mainly arises from mutations in the β-globin gene, which lead to a reduction or complete absence of β-globin synthesis, resulting in an imbalance in the ratio of α to β globin chains and the deposition of excess α-globin responsible for ineffective erythropoiesis and hemolytic anemia [2]. Clinical severities range from asymptomatic carriers with β-thal minor to severe anemia in β-thal intermedia and major, with the latter sometimes requiring lifelong blood transfusions and iron chelation [3].

As reported, β-thal predominantly manifests in the Mediterranean region, the Middle East, India, and Southeast Asia [4, 5]. Recent epidemiological data reveal that carriers of β-thal account for approximately 3% of the global population [6]. Comprehensive studies have shown that the most common mutation type of β-thal in China are mutations within the β-globin gene, including IVS-II-654 (C > T), CD 41-42 (-TTCT), CD 17 (A > T), hemoglobin E (HbE), and a mutation in the promoter region of the β-globin gene −28 (A > G) [6]. Fujian Province, situated in the southern region of China with a population exceeding 40 million permanent residents, exhibits a notably high incidence rate of β-thal. The paucity of large-sample studies on the molecular spectrum and hematological parameters of β-thal in Fujian Province limit our understanding of the disease in this region. A large-scale comprehensive analysis of the correlation between age, gender, genotype, and hematological parameters in Fujian Province was conducted for the first time in this study, potentially providing additional data for genetic counseling and prenatal diagnosis to reduce the incidence of babies born with major thalassemia.

## 2. Methods

### 2.1. Ethical Statement

This study was approved by the institutional ethics committee of Fujian Maternity and Child Health Hospital (approval no. 073, 2019). Signed informed consent was obtained from all participants following a detailed description of the purpose of the study. All experiments were performed by relevant guidelines and regulations.

### 2.2. Study Subjects

From January 2019 to November 2021, a total of 10,350 individuals suspected of being thalassemia carriers were recruited at Fujian Maternity and Child Health Hospital College of Clinical Medicine for Obstetrics and Gynecology and Pediatrics. Those meeting one or more of the following inclusion criteria were screened for the thalassemia gene as (1) mean corpuscular volume (MCV) of < 80 fL and/or mean corpuscular hemoglobin (MCH) concentration of < 27 pg; (2) hemoglobin A2 (HbA_2_) of > 3.5% and/or fetal hemoglobin (HbF) of > 2.0%; and (3) a family history of hereditary thalassemia.

### 2.3. Genotype of β-Thal

DNA was extracted using the DNA Blood Extraction Kit (Yaneng Biosciences, Shenzhen, China), and 17 common β-thal point mutations in the Chinese population were detected by reverse dot blotting (RDB) using a commercial kit (Yaneng Biosciences, Shenzhen, China). Suspected rare types of β-thal were analyzed by amplifying the full-length β-globin gene using PCR, with purified PCR products sequenced directly on an ABI 3100 DNA sequencer (Applied Biosystems; Foster City, CA, USA). The specific methods for these experiments are described in previous literature [4], and all experiments were performed with three replicates to ensure the accuracy and reproducibility of the data.

β^+^-thal means that the mutation leads to a reduction in β-globin chain synthesis; if it leads to a complete failure of β-globin chain synthesis, it is called β^0^-thal. In this study, the β^0^ group contains CD 41-42 (-TTCT)/N, CD 17 (A > T)/N, CD 27/28 (+C)/N, CD 71 -72 (+A)/N, CD 43 (G > T)/N, initiation codon (ATG > AGG)/N, and IVS-I-1 (G > T)/N; the β^+^ group includes IVS-II-654 (C > T)/N, −28 (A > G)/N, IVS-I-5 (G > C)/N, and Cap+40-43 (-AAAC)/N.

### 2.4. Statistical Analysis

The data analysis was conducted using SPSS Version 26 (IBM Inc., Chicago, USA). Normality was assessed with the Kolmogorov–Smirnov test, and variance homogeneity was tested using Levene's test. Parametric tests (ANOVA or *t*-test) were utilized for normally distributed and homogeneous data, while the Kruskal–Wallis test was applied for nonhomogeneous data. Continuous variables are expressed as mean ± standard deviation (SD), and categorical variables are presented as frequencies and percentages. Statistical significance was set at *p* < 0.05 for all tests, with effect sizes calculated to measure the strength of observed differences.

## 3. Results

### 3.1. The Spectrum of β-Thal in Fujian Province

Among the 10,350 subjects screened for suspected thalassemia, 1214 cases were diagnosed as β-thal carriers, with a detection rate of 11.73% (1214/10,350). There were 1148 cases (94.56%) with heterozygotes for β-thal, and 66 cases with concurrent α- and β-thal (5.44%). In this study, 17 kinds of β-thal mutations were identified, the most common genotypes were IVS-II-654 (C > T)/N and CD 41-42 (-TTCT)/N, with a remarkable proportion of 37.56% and 30.72%, and the other common genotypes were CD 17 (A > T)/N (9.64%), −28 (A > G)/N (7.00%), CD 27-28 (+C)/N (3.21%), and CD 26 (GAG > AAG)/N (3.05%). The above genotypes accounted for 91.18% of all β-thal genotypes (Table 1).

Among the 66 cases of concurrent α- and β-thal, 95.45% of the genotypes consisted of common deletions of the α‐globin gene (–SEA/αα, −α3.7/αα, −α4.2/αα) combined with a β‐globin gene point mutation, and composite –SEA/αα and CD 41/42 (‐TTCT)/N was the most common genotype (Table 1).

### 3.2. Rare Types of β-Thal and Their Hematological Parameters

**Figure 1:**
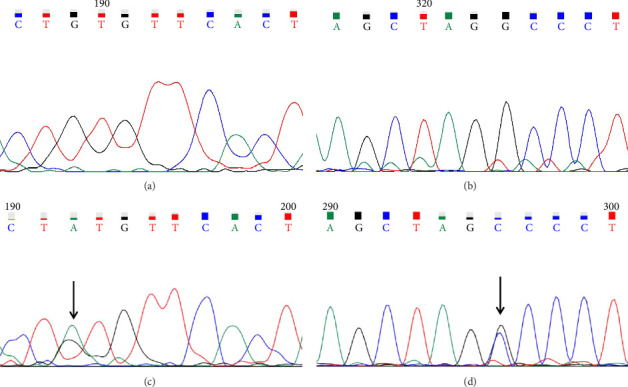
β-Globin gene sequencing variations: comparative analysis of wild-type and mutant alleles. (a) Wild type 1 (WT1). (b) Mutation 1: CAP +22 (G > A). (c) Wild type 2 (WT2). (d) Mutation 2: IVS-II-806 (G > C).

A case with Hb 142 g/L, MCV 63.9 fL, MCH 22.1 pg, and an elevated HbA2 level of 3.8% was identified, suspecting the individual to be a carrier of β-thal. The routine test results appeared normal. Subsequent sequencing of the full-length β-globin gene revealed a double peak at nt192, G > A, which upon comparison was confirmed as the CAP +22 (G > A) mutation (Figures 1(a) and 1(b)). Three additional cases exhibiting elevated HbA2 or HbF levels underwent sequencing of the full-length β-globin gene, revealing a mutation in the intron 2 region, characterized as G > C. This mutation was subsequently confirmed by comparison as IVS-II-806 (G > C) (Figures 1(c) and 1(d)).

In this study, the hematological indices of cases with mutations in the cap locus of the β-globin gene and IVS-II-806 were further summarized. Subject with Cap +22 (G > A)/N exhibited hypochromia and microcytosis (Hb 142 g/L, MCV 63.9 fL, and MCH 22.1 pg) and elevated levels of HbA_2_ (3.8%) and subject with Cap +40-43 (-AAAC)/N showed normal or mild microcytosis with normal HbA_2_ level (Table 2). Two cases with IVS-II-806 (G > C)/N had normal or mild microcytosis with elevated HbA_2_ level (Hb 126 g/L, MCV 78.9 fL, MCH 27.7 pg, and HbA_2_ 3.6%; and Hb 122 g/L, MCV 80.7 fL, MCH 29.0 pg, and HbA_2_ 3.8%). Another subject was diagnosed with double heterozygous IVS-II-806 (G > C) and Hb New York, compound -α4.2/αα mutation, and Hb Q-Thailand and presented mild microcytic hypochromic anemia with decreased HbA_2_ level, elevated HbF level, and detectable level of Hb New York (Hb 121 g/L, MCV 73.8 fL, MCH 24.8 pg, HbA_2_ 2.1%, HbF 17.6%, and Hb New York 28.2%) (Table 3).

### 3.3. Hematological Parameters in β-Thal Carriers of Common Genotypes

In the cohort with β‐thal, the CD 26 group had higher levels of Hb, MCV, and MCH compared to the β^0^ carrier group and the β^+^ carrier group (125.84 ± 12.29 g/L vs. 108.99 ± 14.37 g/L and 106.99 ± 17.08 g/L, 75.78 ± 4.18 fL vs. 63.40 ± 6.41 fL and 63.44 ± 7.86 fL, and 25.49 ± 1.60 pg vs. 20.54 ± 3.02 pg and 20.34 ± 3.0 pg) (*p* < 0.05). As expected, the β^+^ carrier group had higher levels of Hb (108.99 ± 14.37 g/L) compared to the β^0^ carrier group (Hb 106.99 ± 17.08 g/L) (*p*=0.0029) (Table 4).

Further analysis was conducted on the impact of gender and age on the hematological parameters of patients with β-thal, categorizing individuals below 18 years old as immature and those aged 18 and above as adults. It was observed that adult male patients in the β^+^ group had statistically higher levels of Hb compared to adult female patients (131.10 ± 10.75 g/L vs. 102.96 ± 10.44 g/L). Similar results were shown in the β^0^ group (132.08 ± 8.50 g/L vs. 106.46 ± 11.44 g/L). Moreover, both in the β^+^ group and the β^0^ group, adult males had statistically higher levels of Hb, MCV, and MCH compared to immature males (131.10 ± 10.75 g/L vs. 104.09 ± 10.42 g/L, 63.64 ± 2.38 fL vs. 58.83 ± 4.62 fL, and 19.91 ± 0.85 pg vs. 19.27 ± 1.91 pg and 132.08 ± 8.50 g/L vs. 102.66 ± 16.00 g/L, 64.45 ± 4.27 fL vs. 58.22 ± 7.79 fL, and 20.29 ± 1.65 pg vs. 18.73 ± 3.11 pg) (*p* < 0.05). Moreover, both in the β^+^ group and the β^0^ group, adult females had higher MCV and MCH than the immature females (64.34 ± 3.88 fL vs. 60.72 ± 6.71 fL and 20.55 ± 1.70 pg vs. 19.67 ± 2.84 pg and 65.66 ± 5.25 fL vs. 60.04 ± 6.26 fL and 21.08 ± 1.63 pg vs. 19.26 ± 1.96 pg) (*p* < 0.05). However, there was no obvious difference between adult female patients and immature female patients in Hb levels both in the β^+^ group and the β^0^ group (102.96 ± 10.44 g/L vs. 103.27 ± 11.13 g/L and 106.46 ± 11.44 g/L vs. 104.11 ± 10.05 g/L) (*p* > 0.05) (Supporting Table 1).

The hematological parameters of different genotypes within the β^+^ and β^0^ groups were analyzed, focusing on those with case numbers exceeding three. Further comparisons were made between the data of subjects with the −28 (A > *G*)/N mutation and those with IVS-II-654 (C > T)/N in the β^+^ group, as well as between subjects with CD 17 (A > T)/N, CD 71-72 (+A)/N, CD 41-42 (-TTCT)/N, CD 27-28 (+C)/N, and CD 43 (G > T)/N in the β^0^ group. In the β^+^ carrier group, subjects with −28 (A > G)/N had statistically higher levels of Hb, MCV, and MCH compared with IVS-II-654 (C > T)/N (110.62 ± 13.13 g/L vs. 107.82 ± 14.31 g/L, 67.08 ± 3.90 fL vs. 62.13 ± 5.95 fL, and 21.61 ± 1.44 pg vs. 20.12 ± 3.05 pg) (*p* < 0.05), and in the β^0^ carrier group, subjects with CD 17 (A > T)/N, CD 71-72 (+A)/N, CD 41-42 (-TTCT)/N, CD 27-28 (+C)/N, CD 43 (G > T)/N, and initiation codon (ATG > AGG)/N had no statistically significant difference (*p* > 0.05) (Supporting Table 2).

## 4. Discussion

Thalassemia is a globally prevalent hereditary hemolytic disease and is highly prevalent in Southern China, such as Guangxi, Guangdong, Hainan, and Fujian [1]. According to the molecular epidemiological survey of thalassemia in Fujian Province in 2019, the prevalence of β-thal in Fujian was 1.87% [7]. However, there are few studies on the molecular spectrum of β-thal in Fujian Province based on a large sample, and this is the first large-scale comprehensive analysis of the correlation between age, gender, genotype, and hematological parameters in Fujian Province.

In this study, β-thal genes were detected in 1214 out of 10,350 individuals (11.73%) suspected of having thalassemia. Of these, 17 kinds of β-thal mutations were identified, of which the most common mutation types were IVS-II-654 (C > T) (37.56%), CD 41-42 (-TTCT) (30.72%), CD 17 (A > T) (9.64%), −28 (A > G) (7.00%), CD 27-28 (+C) (3.21%), and CD 26 (GAG > AAG) (3.05%) accounting for 91.18% of all mutation types, indicating a high genetic heterogeneity of β-thal in Fujian Province, which was similar to our previous studies [7]. The most common gene mutation types of β-thal in the Fujian Province were IVS-II-654 (C > T) and CD 41-42 (-TTCT), which were different from the reports from Guangxi [8], Guangdong [9], and Hainan [10], where the most common gene mutation was CD 41-42 (-TCTT). In Chongqing [11], the most common gene mutation was CD 17 (A > T), indicating that there was an obvious geographical variation in the gene mutation of β-thal.

Two rare mutations were also detected in this study: Cap +22 (G > A) and IVS-II-806 (G > C). The Cap +22 (G > A) mutation was first identified in Turkey [12] and reported for the first time in China in 2013 by Huang et al. [13]. The 5′UTR of the β-globin gene is about 50 bp from the Cap site to the translation initiation site, and Cap +40-43 (-AAAC), Cap +1 (A > C), +10 (-T), +20 (C > T), +22 (G > A), +33 (C > G), +45 (G > C), and other mutations have been reported [14]. These point mutations generally do not affect the coding sequence of β-globin but affect the transcription level of the β-globin gene [15]. Upon analyzing the hematological parameters of mutations Cap +22 (G > A) and Cap +40-43 (-AAAC), it was observed that heterozygous cases with Cap locus mutations exhibited mild or no significant symptoms of anemia. However, in contrast to cases with the Cap +40-43 (-AAAC) mutation, those with the Cap +22 (G > A) mutation displayed characteristics of hypochromia and microcytosis (MCV 63.9 fL and MCH 22.1 pg), along with an elevated level of HbA2 (3.8%). This suggests that the Cap +22 (G > A) mutation may have a certain inhibitory effect on the transcriptional activity of the β-globin gene, leading to a decrease in the synthesis of β-globin chains and thus affecting the morphology and function of red blood cells. Furthermore, while some studies consider mutations at the Cap site to be merely polymorphisms without significant genetic effects, our research results indicate an association between the Cap +22 (G > A) mutation and anemia phenotype. This finding emphasizes the importance of in-depth research on rare mutations to better understand their role in the pathology of β-thalassemia [16].

The IVS-II-806 (G > C) mutation was first reported in China by Chen et al. in 2020 [17]. The mutation has not been included in the HbVar database and its clinical significance is unknown. In this study, three cases of IVS-II-806 (G > C) mutation were identified, of which two were the only heterozygous mutations of IVS-II-806 (G > C), with hematological parameters such as Hb, MCV, and MCH within normal ranges and slightly elevated HbA2. This is not completely consistent with the three reported blood parameters in the literature, indicating that the phenotype expression of IVS-II-806 (G > C) mutation varies under different genetic backgrounds or environmental factors. This emphasizes the necessity of further studying the molecular mechanism and clinical significance of this mutation under different conditions [18]. The genotype of a double heterozygous individual carrying the IVS-II-806 (G > C) mutation along with Hb New York and compound -α4.2/αα mutation, as well as Hb Q-Thailand, was reported for the first time. This case presented with Hb 121 g/L, MCV 73.8 fL, MCH 24.8 pg, HbA2 2.1%, HbF 17.6%, and Hb New York 28.2%, indicative of mild microcytic hypochromic anemia. Moreover, this case had high levels of HbF, which may be due to the presence of the Hb Q-Thailand mutation, which co-migrates with HbF in capillary electrophoresis [19]. And as this case also carries the Hb-New York mutation, it had an abnormal Hb in zone 11 of capillary electrophoresis [20]. Therefore, compared with IVS-II-806 (G > C)/N cases, cases with IVS-II-806 (G > C) mutations combined with abnormal Hb mutations have lower HbA2 levels and higher HbF levels. These differences may have significant implications for the diagnosis and clinical management of β-thal, highlighting the necessity to account for genetic heterogeneity among individuals in clinical practice.

The clinical symptoms of patients with β-thal vary in severity, but most of them are characterized by chronic progressive hemolytic anemia with altered hematological phenotypes such as Hb, MCV, MCH, MCHC, and HbA_2_ concentrations [21]. Different mutation types of the β-globin gene have different effects on the function of the β-globin gene, β^+^-thal means that the mutation type leads to a reduction in β-globin chain synthesis, and if it leads to a complete failure of β-globin chain synthesis, it is called β^0^-thal. In the present study, the hematological phenotypes of β^0^-thal, β^+^-thal, and CD 26 were analyzed, showing that subjects with β^0^-thal had statistically lower levels of Hb and MCV than subjects with β^+^-thal (*p* < 0.05), while the subjects with CD 26 (GAG > AAG) mutation had higher levels of Hb, MCV, and MCH compared to the β^0^ carriers and the β^+^ carriers, which is consistent with previous studies [22]. CD 26 (GAG > AAG) refers to the mutation of glutamate to lysine at position 26 of the β-globin peptide chain, which reduces the amount of β-globin gene synthesis but synthesizes HbE that is still functional with abnormal structure and reduces the degree of anemia in patients [23]. Therefore, patients with mutation type 26 (GAG > AAG)/N, who have a clinical phenotype of β^+^-thal, have little change in their hematological phenotype and very mild or even no significant symptoms of anemia. However, patients who co-inherited severe β^+^ or β^0^-thal alleles might be more severely affected [24].

This study evaluated the effect of age, gender, and genotype on phenotype in β-thal and found that in the β^+^ carrier group, subjects with −28 (A > G)/N had statistically higher levels of Hb, MCV, and MCH compared with IVS-II-654 (C > T)/N, and in the β^0^ carrier group, subjects with CD 17 (A > T)/N, CD 71-72 (+A)/N, CD 41-42 (-TTCT)/N, CD 27-28 (+C)/N, and CD 43 (G > T)/N had no statistically significant difference (*p* > 0.05). Interestingly, our study revealed that adult male patients with β-thal had significantly higher Hb levels than adult female patients, a phenomenon observed in both the β^+^ and β^0^ groups. This discrepancy might be attributed to gender-related physiological differences, such as the significant increase in testosterone levels during male puberty (*p* < 0.05), which promoted the development of muscles and bones, as well as the enhancement of cardiopulmonary function, leading to generally higher Hb levels in males compared to females [25].

In this study, MCV and MCH levels in adult β-thal individuals were found to be significantly higher than those in immature individuals (*p* < 0.05), indicating a maturation effect on hematological parameters with age, consistent with trends seen in healthy populations [26, 27]. However, the difference in Hb levels between adult and immature females was not statistically significant (*p* > 0.05), which was inconsistent with results from healthy populations [28]. This might be due to the significant participation of pregnant individuals in this study and during pregnancy, the increase in blood volume led to the dilution of Hb, resulting in lower Hb levels [29, 30]. These findings suggest that clinicians should consider gender and age factors when assessing hematological indicators in β-thal patients to diagnose and monitor the disease state more accurately. The results of the study underscore the importance of establishing customized reference ranges for β-thal patients, especially for children and immature patients, to prevent underdiagnosis and misdiagnosis.

While this study presents a comprehensive molecular epidemiological investigation and hematological analysis within a substantial sample population from Fujian Province, several limitations must be acknowledged. Firstly, the generalizability of our findings to the broader Chinese population or other regions may be constrained by the geographic specificity of the sample cohort. Secondly, our research, which concentrated on molecular and hematological parameters, did not account for potential influencing factors such as environmental aspects and lifestyle habits. Additionally, although we considered variables like gender, age, and genotype, there may be unmeasured confounding factors, including the patients' overall health status and lifestyle, that could influence hematological parameters. Nonetheless, our investigation offers novel insights by elucidating the complex interplay among age, gender, genotype, and hematological parameters within the β-thal phenotype in Fujian Province. The innovative aspect of our study is its detailed analysis of a large dataset, yielding region-specific understanding of β-thal's molecular epidemiology. We are confident that our findings will enhance diagnostic and therapeutic strategies for β-thal in Fujian and may provide valuable insights for other regions.

## 5. Conclusion

### 5.1. Comprehensive Molecular Epidemiological Investigation and Hematological Analysis

This study represents the first comprehensive molecular epidemiological investigation and hematological analysis of β-thal in Fujian Province, a significant contribution toward optimizing thalassemia prevention and control strategies.

### 5.2. Novel Findings in Hematological Parameters and Genotype

Notably, this study reports for the first time the hematological parameters and genotype in a patient with compound heterozygosity of Hb New York and IVS-II-806 (G > C) mutation with −α4.2/αα and Hb Q-Thailand. This finding offers valuable insights for prenatal genetic counseling.

This study also reveals that gender and age are the key factors affecting the hematological parameters of β-thal patients and emphasizes that the individualized differences of gender and age shall be considered in the clinical diagnosis and treatment of β-thal.

## Figures and Tables

**Table 1:** Genotype frequency distribution of β-thal in the Fujian population.

	Genotype of β-thal	*N*	Percentage in β-thal (%)	Detected rate (%)
β-thal	β^IVS-II-654^^(C>T)^/β^N^	456	37.56	4.41
	β^CD41-42 (-TTCT)^/β^N^	373	30.72	3.60
	β^CD17 (A>T)^/β^N^	117	9.64	1.13
	β^−28^^(A>G)^/β^N^	85	7.00	0.82
	β^CD27-28 (+C)^/β^N^	39	3.21	0.37
	β^CD26 (GAG>AAG)^/β^N^	37	3.05	0.35
	β^CD71 -72^^(+A)^/β^N^	15	1.24	0.14
	β^CD43 (G>T)^/β^N^	12	0.99	0.12
	β^Initiation codon (ATG>AGG)^/β^N^	4	0.33	0.04
	β^IVS-II-806^^(G>C)^/β^N^	2	0.16	0.04
	β^Cap+40-43 (-AAAC)^/β^N^	2	0.16	0.02
	β^Cap+22 (G>A)^/β^N^	1	0.08	0.01
	β^IVS-I-1^^(G>T)^/β^N^	1	0.08	0.01
	β^IVS-I-5^^(G>C)^/β^N^	1	0.08	0.01
	β^CD30 (AGG>GGG)^/β^N^	1	0.08	0.01
	β^−29^^(A>G)^/β^N^	1	0.08	0.01
	β^CD14-15 (+G)^/β^N^	1	0.08	0.01

Subtotal		**1148**	**94.56**	**11.24**

Concurrent α- and β-thal	-α3.7/αα, β^IVS-II-654^^(C>T)^/β^N^	11	0.90	0.11
	-α3.7/αα, β^CD41-42 (-TTCT)^/β^N^	4	0.33	0.03
	-α3.7/αα, β^CD71 -72^^(+A)^/β^N^	2	0.16	0.02
	-α3.7/αα, β^CD17 (A >T)^/β^N^	2	0.16	0.02
	-α3.7/αα, β^−28^^(A>G)^/β^N^	1	0.08	0.01
	-α3.7/αα, β^Cap+40-43 (-AAAC)^/β^N^	1	0.08	0.01
	-α3.7/-- SEA, β^CD41-42 (-TTCT)^/β^N^	1	0.08	0.01
	-α4.2/αα, β^CD41-42 (-TTCT)^/β^N^	2	0.16	0.02
	-α4.2/αα, β^CD17 (A>T)^/β^N^	1	0.08	0.11
	-α4.2/αα/Hb Q Thailand, β^IVS-II-806^^(G>C)^/β^N^/Hb New York	1	0.08	0.11
	--SEA/αα, β^IVS-II-654^^(C>T)^/β^N^	11	0.90	0.13
	--SEA/αα, β^CD41-42 (-TTCT)^/β^N^	12	0.98	0.04
	--SEA/αα, β^−28^^(A>G)^/β^N^	4	0.33	0.03
	--SEA/αα, β^CD27-28 (+C)^/β^N^	3	0.24	0.03
	--SEA/αα, β^CD17 (A>T)^/β^N^	3	0.24	0.03
	--SEA/αα, β^Cap+40-43 (-AAAC)^/β^N^	1	0.08	0.01
	--SEA/αα, β^CD26 (GAG>AAG)^/β^N^	1	0.08	0.01
	--SEA/-α3.7, β^IVS-II-654^^(C>T)^/β^N^	1	0.08	0.01
	--SEA/-α4.2, β^CD41-42 (-TTCT)^/β^N^	1	0.08	0.01
	αα^CS^/αα, β^CD17 (A>T)^/β^N^	1	0.08	0.01
	αα^CS^/αα, β^IVS-II-654^^(C>T)^/β^N^	1	0.08	0.01
	αα^WS^/αα, β^IVS-II-654^^(C>T)^/β^N^	1	0.08	0.01

Subtotal		**66**	**5.44**	**0.63**

Total		**1214**	**100.00**	**11.87**

*Note: N* indicates no mutation. SEA, Southeast Asian deletion; THAI, Thailand deletion; WS, Hb Westmead. The bolded sections, “Subtotal” and “Total,” summarize key data on the distribution of β-thalassemia genotypes in the Fujian population. The “Subtotal” sections provide specific insights into the distribution of simple β-thalassemia and concurrent α- and β-thalassemia genotypes within the β-thalassemia population. These figures are crucial for understanding the prevalence of different β-thalassemia types and their detection rates in the studied sample. Meanwhile, the “Total” section offers a comprehensive overview of all β-thalassemia genotypes, giving us the overall incidence and detection rate of β-thalassemia in the entire population. These bolded summaries are essential for grasping the main findings and implications of the study.

**Table 2:** Hematological parameters of heterozygous cases with mutations in the cap locus of the β-globin gene.

Genotype	Age	Gender	Hb (g/L)	MCV (fL)	MCH (pg)	HbA_2_ (%)	HbF (%)
β^Cap+22 (G>A)^/β^N^	28	Male	142	63.9	22.1	3.8	0.5
β^Cap+40-43 (-AAAC)^/β^N^	31	Female	120	79.3	24.9	2.9	/
β^Cap+40-43 (-AAAC)^/β^N^	29	Male	138	88.7	29.9	2.8	/

**Table 3:** Hematological parameters associated with IVS-II-806 (G > C) mutation cases.

Genotype	Age	Gender	Hb (g/L)	MCV (fL)	MCH (pg)	HbA_2_ (%)	HbF (%)
β^IVS-II-806 (G>C)^/β^N^	27	Female	126	78.9	27.7	3.6	0.4
β^IVS-II-806 (G>C)^/β^N^	24	Female	122	80.7	29.0	3.8	0.0
-α4.2/αα/Hb Q, β^IVS-II-806(G>C)^/β^N^/Hb New York	33	Female	121	73.8	24.8	2.1	17.6

**Table 4:** Hematological parameters in thalassemia carrier of common genotype.

	Hb (g/L)	MCV (fL)	MCH (pg)
β^CD26(GAG>AAG)^/β^N^	125.84 ± 12.29	75.78 ± 4.18	25.49 ± 1.60
β^+^/β^N^	108.99 ± 14.37	63.40 ± 6.41	20.54 ± 3.02
β^0^/β^N^	106.99 ± 17.08	63.44 ± 7.86	20.34 ± 3.08
*p* value^a^	< 0.0001^∗∗∗∗^	< 0.0001^∗∗∗∗^	0.0110^∗^
*p* value^b^	< 0.0001^∗∗∗∗^	< 0.0001^∗∗∗∗^	0.0076^∗∗^
*p* value^c^	0.0029^∗∗^	0.9976	0.9421

^a^Comparison between subjects with β^CD26(GAG>AAG)^/β^N^ and those with β^+^/β^N^.

^b^Comparison between subjects with β^CD26(GAG>AAG)^/β^N^ and those with β^0^/β^N^.

^c^Comparison between subjects with β^+^/β^N^ and those with β^0^/β^N^.

^∗^ *p* < 0.05.

^∗∗^ *p* < 0.01.

^∗∗∗∗^ *p* < 0.0001, Kruskal–Wallis test.

## Data Availability

The datasets used and/or analyzed during the current study are available from the corresponding authors on reasonable request.

## Supporting Information

Supporting Table 1: the table shows hematological parameters of β-thal in different genders. Supporting Table 2: the table shows hematological parameters in β-thal carrier of common genotype.
