# Supplementary material for: Fujian Province β-Thalassemia: A Molecular and Hematological Study in Southeastern China
Source: Genet Res (Camb). 2025 Jun 8;2025:8862095. doi: 10.1155/genr/8862095 (PMC12168651; doi:10.1155/genr/8862095)
Supplement: Supporting Information — Additional supporting information can be found online in the Supporting Information section. [file 8862095.f1.docx]

**Supplemental Tables**

Supplemental Table 1 Hematological parameters of β-thalassemia in different gender and age

|  |  | **0-18years old** | | | **>18years old** | | |
| --- | --- | --- | --- | --- | --- | --- | --- |
|  | **Gender** | Hb(g/L) | MCV(fl) | MCH(pg) | Hb(g/L) | MCV(fl) | MCH(pg) |
| **β^+^** | Male | 104.09 ±10.42 | 58.83 ±4.62 | 19.27 ±1.91 | 131.10±10.75 | 63.64±2.38 | 19.91±0.85 |
|  | Female | 103.27 ±11.13 | 60.72 ±6.71 | 19.67±2.84 | 102.96±10.44 ^a^ | 64.34±3.88 | 20.55±1.70 |
| **β^0^** | Male | 102.66 ±16.00 | 58.22 ±7.79 | 18.73 ±3.11 | 132.08±8.50 | 64.45±4.27 | 20.29±1.65 |
|  | Female | 104.11±10.05 | 60.04 ±6.26 | 19.26 ±1.96 | 106.46±11.44 | 65.66±5.25 | 21.08±1.63 |

Supplemental Table 2 Hematological parameters in β-thalassemia carrier of common genotype

|  | **Genotype** | **Hb(g/L)** | **MCV(fl)** | **MCH(pg)** |
| --- | --- | --- | --- | --- |
| **β^+^/β^N^** | β^-28 ( A>G)^/β^N^ | 110.62±13.13 | 67.08±3.90 | 21.61±1.44 |
|  | β^IVS-II-654 (C>T)^/β^N^ | 107.82±14.31 | 62.13±5.95 | 20.12±3.05 |
| **β^0^/β^N^** | β^CD 17 (A > T)^/β^N^ | 108.50±20.64 | 64.60±9.96 | 20.77±3.78 |
|  | β^CD 71 -72 ( +A)^/β^N^ | 108.60±14.06 | 61.95±5.26 | 19.88±1.64 |
|  | β^CD 41-42 ( -TTCT)^/β^N^ | 106.74±16.70 | 63.19±7.48 | 20.28±2.99 |
|  | β^CD 27-28( +C)^/β^N^ | 105.19±10.42 | 63.52±5.33 | 20.12±2.13 |
|  | β^CD 43 ( G > T)^/β^N^ | 100.25±6.45 | 63.38±4.43 | 19.65±1.58 |
|  | β^Initiation codon (ATG>AGG)^/β^N^ | 101.5±6.26 | 57.275±4.56 | 17.75±0.78 |
| *P‐value ^1^* | | <0.001^*^ | <0.001^*^ | <0.001^*^ |
| *P‐value ^2^* | | >0.9999 | >0.9999 | >0.9999 |

^1^ *P‐value* between subjects with -28 ( A>G)/N and IVS-II-654( C>T)/N.

^2^ *P‐value* between subjects with CD 17 ( A>T)/N, CD 71 -72 ( +A)/N ,CD 41-42 ( -TTCT)/N, CD 27 -28 ( +C)/N, CD 43( G>T)/N, Initiation codon (ATG>AGG)/N.

*P<0.05,  Kruskal‐Wallis test.
